# Genotypes and Antibiotic Resistances of *Campylobacter jejuni* Isolates from Cattle and Pigeons in Dairy Farms

**DOI:** 10.3390/ijerph110707154

**Published:** 2014-07-14

**Authors:** Valentina Bianchini, Mario Luini, Laura Borella, Antonio Parisi, Romie Jonas, Sonja Kittl, Peter Kuhnert

**Affiliations:** 1 Istituto Zooprofilattico Sperimentale della Lombardia e dell’Emilia Romagna, Lodi 26900, Italy; E-Mails: bianchini.valentina87@gmail.com (V.B.); laurabl83@gmail.com (L.B.); 2 Istituto Zooprofilattico Sperimentale della Puglia e della Basilicata, Putignano (BA) 70017, Italy; E-Mail: parisi.izspb@gmail.com; 3 Institute of Veterinary Bacteriology, Vetsuisse Faculty, University of Bern, Bern CH-3001, Switzerland; E-Mails: r.jonas987@gmail.com (R.J.); sonja.kittl@vetsuisse.unibe.ch (S.K.); peter.kuhnert@vetsuisse.unibe.ch (P.K.)

**Keywords:** *Campylobacter jejuni*, dairy farms, pigeons, genotyping, antibiotic resistance

## Abstract

*Campylobacter jejuni* is the most common food-borne zoonotic pathogen causing human gastroenteritis worldwide and has assumed more importance in Italy following the increased consumption of raw milk. Our objectives were to get an overview of genotypes and antibiotic resistances in *C. jejuni* isolated from milk, cattle feces, and pigeons in dairy herds of Northern Italy. *flaB*-typing was applied to 78 *C. jejuni* isolates, previously characterized by Multi-Locus Sequence Typing, and genotypic resistances towards macrolides and quinolones based on point mutations in the 23S rRNA and *gyrA* genes, respectively, were determined. *flaB*-typing revealed 22 different types with one of them being novel and was useful to further differentiate strains with an identical Sequence Type (ST) and to identify a pigeon-specific clone. Macrolide resistance was not found, while quinolone resistance was detected in 23.3% of isolates. A relationship between specific genotypes and antibiotic resistance was observed, but was only significant for the Clonal Complex 206. Our data confirm that pigeons do not play a role in the spread of *C. jejuni* among cattle and they are not responsible for milk contamination. A relevant number of bulk milk samples were contaminated by *C. jejuni* resistant to quinolones, representing a possible source of human resistant strains.

## 1. Introduction

*Campylobacter jejuni* asymptomatically colonize the intestine of many food-producing animals, but also wildlife [1]. *C. jejuni* is the most frequent cause of bacterial gastroenteritis in humans [2] and has gained more importance in Italy following the increased consumption of raw milk. Human Campylobacteriosis is usually a self-limiting disease and treatment is often limited to maintenance of hydration. Antimicrobial therapy is needed only for severe infections and in immunocompromised patients. Currently, macrolides are the most common antimicrobial agents prescribed when a therapeutic intervention is required. Quinolones are also indicated as the first-line therapy since they are the drugs of choice for empirical treatment of acute bacteria diarrhea [3]. The indiscriminate use of antibiotics in humans, as well as the use of antimicrobials in livestock for disease prevention and control, has led to an increased antibiotic-resistance in *Campylobacters*, particularly with regard to quinolones and macrolides [4]. Quinolone resistance in *C. jejuni* occurs via specific point mutations in the quinolone resistance-determining region of the *gyrA* gene, with the C257T mutation being the most common. High-level macrolide resistance is conferred by point mutations in the peptidyl transferase region in domain V of the 23S rRNA gene at positions 2074 or 2075 [5,6].

We previously investigated the presence of *C. jejuni* in bulk tank milk of 282 dairy herds in the Lodi Province and the microorganism was detected in 12% of the examined samples. In three *C. jejuni*-positive farms we also examined the presence of the microorganism in bovine feces and pigeons to evaluate their role in milk contamination. We found that fecal excretion was common in milking cows (30.5%) and pigeons were frequently colonized by *C. jejuni* (21.7%) [7]. Seventy-eight *C. jejuni* strains isolated from bulk tank milk, bovine feces, and pigeons were genotyped by Multi-Locus Sequence Typing (MLST) and this analysis revealed lineages common between milk and bovine feces, but distinct between cattle and pigeons, suggesting that bovine feces could be responsible for the presence of the pathogen in milk. On the other hand, pigeons probably do not play a role in the transmission of *C. jejuni* to cattle and in milk contamination. We also described some cases of milk contamination due to chronic udder infection and this alternative way of milk contamination should be taken into account especially when *C. jejuni* is demonstrated repeatedly [7].

MLST is the method of choice to study the epidemiology of *Campylobacter* [8]. Sequencing of the short variable region within the flagellin-encoding gene *flaB* slightly increases the discriminatory power of MLST, allowing a more precise differentiation among some strains that have the same MLST sequence type [9].

The aim of the study was to further characterize *C. jejuni* strains isolated in Northern Italy from bulk tank milk, cattle, and pigeons. In particular, *flaB*-typing and sequence-based determination of quinolone and macrolide resistances were used to address the following aspects: (i) the diversity of *C. jejuni* isolated from milk, bovine feces and pigeons; (ii) the possible role of cattle and pigeons in milk contamination; (iii) the antibiotic resistance of *C. jejuni* strains; (iv) the possible relationship between specific genotypes and antibiotic resistance/susceptibility.

## 2. Experimental Section

### 2.1. Samples

**Figure 1:**
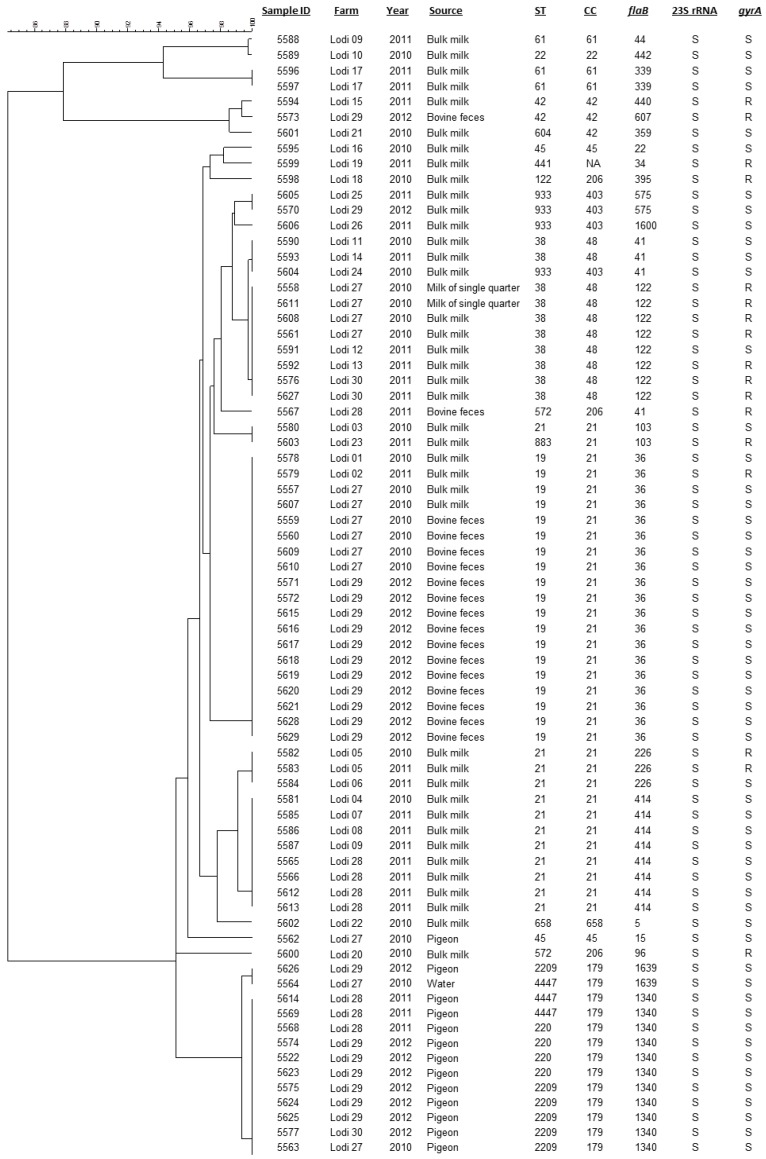
Clustering of the strains based on the partial *flaB* gene sequences. The UPGMA tree was constructed in BioNumerics. For each strain are indicated: farm, year and source of isolation; MLST (ST and CC) and *flaB* type; the susceptibility (S) or resistance (R) to macrolides (23S rRNA) and quinolones (*gyrA*).

A total of 78 *C. jejuni* strains collected between 2010 and 2012 during a survey in the Lodi Province (Lombardy Region, Northern Italy) and previously characterized by MLST [7] were included in the study. They were isolated from 30 dairy herds from as many samples of bulk tank milk and, in four of these farms, also from different sources: bovine feces (*n* = 21), pigeon intestine (*n* = 13), additional bulk tank milk (*n* = 10), milk of single quarter (*n* = 2), and water points (*n* = 2). Information about farms, years, and sources of isolation are indicated in Figure 1.

### 2.2. Genotyping and Determination of Antibiotic Resistance

The strains were characterized by *flaB* sequence-based typing and mutations within the 23S rRNA and *gyrA* genes that confer resistance to macrolide and quinolone antibiotics, respectively, were analyzed based on the sequencing of 23S rRNA and *gyrA* gene fragments. The analyses were performed according to the previously described method developed by Korczak *et al.* [9].

### 2.3. Data Analysis

Sequence data were edited and analyzed using the software Sequencher v5.0 (GenCodes, Ann Arbor, MI, USA) and entered into the BioNumerics program v7.1 (Applied Maths NV, Sint-Martens-Latem, Belgium) for cluster analysis using the unweighted pair group method with arithmetic mean (UPGMA).

The genotypes of *flaB* were determined with a tool that is provided by the PubMLST database [10]. The new genotypes were submitted directly to the curator of the PubMLST database for allele number assignment.

The discriminatory abilities of *flaB* and MLST were calculated using Simpson’s index of diversity [11].

*gyrA* and 23S rRNA amplified fragments were checked for the presence of the following mutations: C257T or A and A256G within the *gyrA* gene and A2074G or C and A2075G within the 23S rRNA gene.

The association of certain genotypes with the presence of quinolone resistance was tested by Fisher’s exact two-tailed test. A *p* value of <0.05 was used to indicate statistically significant results.

## 3. Results and Discussion

### 3.1. Typing

The *flaB* (446 bp), *gyrA* (253 bp) and 23S rRNA (465 bp) target gene fragments could be successfully amplified and their sequence determined for 73 out of the 78 *C. jejuni* strains. In the remaining five cases mixed sequences were obtained for one or more of the target genes.

A total of 22 *flaB* types were obtained, 12 of which were represented by single isolates. One new *flaB* allele (assigned number 1639) was observed in two strains recovered from a pigeon and a water point. The most frequent *flaB* types were 36 (26%), 1340 (15.1%), 41 (11%), and 122 (11%).

Cluster analysis of the sequenced *flaB* fragments revealed a pigeon-specific cluster (Figure 1).

In some cases, *flaB* genotyping allowed further separation of strains that belonged to the same Sequence Type (ST), although different STs can be found with the same *flaB* sequence. Generally, the resolution of the *flaB* sequence-based genotyping is slightly higher than that of MLST [9]. In our study, the *flaB*-typing confirmed the results that were obtained by the MLST analysis and this genotyping technique was useful to further differentiate between certain strains isolated from bulk milk and bovine feces. Indeed, cattle isolates were subdivided into 14 different sequence types by MLST and into 19 *flaB*-types. Interestingly, the *flaB*-type of 11 out of 13 pigeon isolates, which were subdivided into three different STs, resulted the same (type 1340), indicating the existence of a pigeon-specific clone, different from the strains recovered from milk and bovine feces. This is in agreement with the observations that wild birds harbor host specific *C. jejuni* populations clonally different from human and domestic animals [12]. One pigeon strain showed a new *flaB*-type (type 1639), recovered in our study also from a water point, and by cluster analysis these isolates were grouped together with other pigeon isolates. Also by MLST these two strains were included into the pigeon-specific lineage (Clonal Complex 179). Only one pigeon strain belonged to a different MLST profile, ST-45, a genotype found also in bulk milk, but *flaB*-typing indicated that these two strains were different (pigeon isolate was classified as type 15, milk isolate as type 22).

According to these data, *flaB*-typing allowed us to confirm that pigeons are not relevant for the transmission of *C. jejuni* to cattle and for milk contamination. *flaB*-typing was helpful to further differentiate strains with the same ST and provided a slightly greater discriminatory potential than MLST (Simpson’s indexes of diversity for MLST 0.83, Simpson’s indexes of diversity for *flaB*-typing 0.89).

### 3.2. Genotypic Antibiotic Resistance

The investigation on genotypic antimicrobial resistance showed a low prevalence of the genetic determinants we investigated. The C257T or A and the A256G mutations in the *gyrA* gene and the two mutations A2074C and A2075G in the 23S rRNA gene were selected since they are responsible for resistance to the drugs of choice for human campylobacteriosis therapy and they are allowed for use in veterinary medicine in Italy. The mutations in the 23S rRNA gene that contribute to macrolide resistance were not observed. The transition C257T within the *gyrA* gene leading to quinolone resistance was present in 17 isolates (23.3%; 95% confidence interval = 13.6% to 33%). The transition was found in milk and bovine feces strains, but not in *C. jejuni* isolated from pigeon intestines and water point (Table 1).

**Table 1:** Antimicrobial resistance pattern of *C. jejuni* isolates.

Source	No. of Samples	No. of Resistant Isolates (%)	
		Quinolones	Macrolide
Bulk tank milk	40	13 (32.5)	0
Milk of single quarter	2	2 (100)	0
Bovine feces	17	2 (11.8)	0
Pigeon intestine	13	0	0
Water point	1	0	0
Total	73	17 (23.3)	0

An increase in the number of *C. jejuni* and *C. coli* strains that are resistant to frequently used antibiotics (macrolides and, especially, the quinolones) has been reported worldwide [4,13]. In Italy, only partial data on the diffusion of *Campylobacter* are available: human cases notification is based on a voluntary system and there is little information about the antimicrobial resistance pattern of human and bovine *Campylobacter* strains. The European Food Safety Authority (EFSA) reported 75.7 and 6.3% strains resistant to quinolones and macrolides, respectively, in human population in 2011 in Italy. In the same year, the reported percentages of resistant strains in cattle were 56.3 (quinolones) and 2.1 (macrolides) [13]. Lower rates of resistance to quinolones were described in Northeastern (25%) [14] and Southeastern Italy (18%) [15]. In Italy, drugs belonging to quinolones (such as marbofloxacin, enrofloxacin, and danofloxacin) and macrolides (such as tylosin, spiramycin, and tilmicosin) are widely used in veterinary medicine to treat bovine respiratory disease and metritis and mastitis in cattle.

In this study, no *C. jejuni* isolates were macrolide resistant, whereas 23.3% of *C. jejuni* isolates were resistant to quinolones. These data confirm that in Italy antimicrobial resistance rate in *C. jejuni* is moderate, but it should be kept under observation, especially because a relevant number of bulk milk samples (13 out of 40) was contaminated by *C. jejuni* resistant to quinolones, representing a possible risk for human infections with antibiotic resistant strains.

### 3.3. Association between Genotype and Antibiotic Resistance

Since isolates collected on the same farm and showing the same MLST and *flaB*-typing profile also shared the same resistance pattern, they were considered to be related isolates representing the same strain. According to these epidemiological and genetic findings, 43 *C. jejuni* strains (32 from bulk milk, three from bovine feces, seven from pigeon intestine and one from water point) were identified. These data were used to test the possible association of certain genotypes with quinolone resistance. Resistant strains belong to clonal complexes (CC) 21 (*n* = 3), 48 (*n* = 3), 206 (*n* = 3), 42 (*n* = 2), and to sequence type (ST) 441 (*n* = 1). Only CC-206 showed a significant association with quinolone resistance (*p* = 0.02).

Identical genotypes did not necessarily show identical quinolone resistance patterns, although in most cases strains sharing the same genotype displayed an identical genotypic antimicrobial resistant pattern. Other studies reported a relationship between specific genotypes (determined by MLST) and the occurrence of genetic determinants of antimicrobial resistance, particularly resistance to quinolones [16,17]. In our investigation, a significant association with quinolone resistance (*p* = 0.02) was found for CC-206. Additionally, 23.1% of *C. jejuni* CC-21 strains were resistant to quinolones, which was also seen in previous studies [16,17], suggesting that there is a correlation between specific genotypes and antibiotic resistance. Since mutations in the *gyrA* can not only be rapidly acquired but they are actively promoted under treatment [18], they can occur independently in different genotypes as well as in certain strains of a defined ST but not in others. Therefore, a direct association between resistance and a specific genotype is not necessarily given, but rather indicates the rapid spread of a specific clone.

## 4. Conclusions

In conclusion, *flaB*-typing led us to identify a pigeon-specific clone, allowing us to confirm that pigeons do not play a role in the spread of *C. jejuni* among cattle and they are not responsible for milk contamination. In combination with MLST, the *flaB* genes increased the discriminatory power of the method, which could be helpful in certain situations.

Our study provides evidence that quinolones resistance is a common phenomenon in dairy farms in Northern Italy and thus indicates the need for an appropriate strategy of surveillance and epidemiological monitoring to control the development of resistance. The investigation of antimicrobial susceptibility in animal *Campylobacter* is important because the emergence of resistant strains in animals may cause an increase in human infections difficult to treat. On the basis of these results, the implementation of specific control procedures is strongly recommended in order to limit the diffusion of resistant strains.
